# Scalp seborrheic dermatitis demonstrates a skewing of Th1 activation: a proteomic study in lesional skin

**DOI:** 10.3389/fimmu.2025.1638710

**Published:** 2025-09-29

**Authors:** Ningning Shen, Wei Chen, Lihua Hu, Jia Huang, Qiang Dong

**Affiliations:** Department of Dermatology, Dermatology Hospital of Zhejiang Province, Huzhou, Zhejiang, China

**Keywords:** inflammatory, Olink, proteomic, scalp, seborrheic dermatitis

## Abstract

**Introduction:**

Scalp seborrheic dermatitis (SSD) is a common, chronic inflammatory skin disease. Its pathogenesis and immunological features have been poorly studied.

**Objective:**

To elucidate the molecular profile of adult patients with SSD in lesional scalps.

**Methods:**

Using punch biopsies, we assessed 92 inflammatory biomarkers in the lesional scalps of SSD patients (n=16) and demographically matched healthy controls (HCs; n=12) via Olink high-throughput proteomics.

**Results:**

We identified 16 differentially expressed proteins (DEPs) between lesional scalps of patients with SSD and those of HCs. SSD lesional scalps demonstrated significantly greater expressions of proteins related to T-cell/lymphocyte activation, the cytokine storm signaling pathway and the CGAS-STING signaling pathway. Ingenuity pathway analysis (IPA) highlighted Th1 skewing. These data suggest that SSD is associated with Th1 skewing and the dysregulation of lipid metabolism.

**Conclusion:**

These analyses provide a rationale for novel treatment approaches for SSD patients, mainly those targeting Th1 pathways.

## 1 Introduction

Seborrheic dermatitis (SD) is a common, chronic, and inflammatory skin disease characterized by erythematous and scaly plaques. It typically affects skin with abundant sebaceous glands, such as the scalp (1). The overall prevalence of scalp seborrheic dermatitis (SSD) is 3.3%. SSD is more likely to affect young and middle-aged individuals and can negatively influence patient quality of life; it has been demonstrated that there is a higher impact on QoL in males than in females (2, 3). Although a variety of topical or new oral drugs are used, treatment may be limited by efficacy and side effects. In addition, SSD is sometimes difficult to distinguish from scalp psoriasis (SP). These clinical factors suggest that further exploration of the molecular immunological characteristics of this disease is needed.

The pathogenesis of SSD is not entirely clear. Research has shown that SSD involves interactions among skin flora, particularly Malassezia spp., skin surface lipids and personal susceptibility (3, 4). Studies in human skin/scalp biopsies with limited assessments of biomarkers have shown abnormal expression of several innate, T-helper (Th)1, and Th2 molecules, such as tumor necrosis factor-a (TNF-a), interleukin (IL)-1a/IL-1b, IL-4, IL-10, and IL-12 (5, 6). Several transcriptional analyses revealed significant upregulation of expression of IL-23/Th17 and Th22, with some demonstrating Th1 skewing (7), and increased protein expression in the stratum corneum (e.g., of IL-1RA, S100s, and IL-8) (8). Recently, the new Olink proteomic platform has been used to investigate the immunological characteristics of skin diseases. It requires only 10 µg of tissue per sample and can be easily obtained with a 1 mm trephine with minimal trauma. Current studies focus mainly on atopic dermatitis (AD) (9–11), alopecia areata (12), psoriasis and hidradenitis suppurative (HS) (13–15). Most of these studies use blood samples instead of skin samples. Olink platforms used in skin biopsies of SSD have been poorly studied.

Therefore, we aimed to characterize SSD proteomics using the Olink platform in lesional scalps of patients with SSD in comparison to scalps of HCs. Our data provide a rationale for novel treatment approaches for SSD patients.

## 2 Materials and methods

### 2.1 Patient enrollment

This study was approved by the Institutional Review Board of the Dermatology Hospital of Zhejiang Province (Approval NO: Dermatology Hospital of Zhejiang Province-2025 ethical review NO 02K), and written informed consent was obtained. Untreated patients with SSD who were 18 years of age or older (n=16) and demographically matched HCs (n=12) were enrolled in the study. Patients were included if they had not used systemic immunosuppressants, biological agents or phototherapy within three months or local therapeutic drugs within one month. We excluded patients with other inflammatory skin diseases, such as psoriasis and eczema.

### 2.2 Skin sample collection

Participants were assessed and sampled at baseline. SSD lesional punch biopsies (3 mm) were obtained from an active inflammatory lesion. Skin tissues were placed in 5-mL Eppendorf tubes, frozen in liquid nitrogen for 5–10 min, and stored at -80°C.

### 2.3 Skin protein extraction and quantification

#### 2.3.1 Sample lysis and protein extraction

The skin samples were processed by adding an appropriate volume of complete weak RIPA lysis buffer (containing 50 mmol/L Tris-HCl (pH 7.4), 150 mmol/L NaCl, 1% NP-40, 0.25% sodium deoxycholate, sodium orthovanadate, sodium fluoride, EDTA, and leupeptin) to each sample, after which protease inhibitors were added at a 1:1000 volume ratio to prevent degradation. One scoop (0.21 g) of stainless-steel beads was added to each tube, and the tubes were placed in a tissue grinder. The samples were ground at 2°C, and 60 Hz (10 s per cycle, 10 s interval, for a total of 60 cycles) until homogenized. The homogenate was transferred to a noncontact ultrasonic cell disruptor for treatment. Samples were then put in a centrifuge at 12,000–15,000 × g for 15–20 min at 4°C, after which the supernatant was collected (crude skin protein extract).

#### 2.3.2 Determination of protein concentration

The BCA method: Standards and working detection solution were prepared per the instructions of the BCA kit (P0012, Beyotime; Shanghai, China). Standards and diluted crude extract were added to a 96-well plate. The samples were incubated at 37°C for 30 min, after which the absorbance was measured at 562 nm using a microplate reader. A standard curve was generated from the standard absorbance, and the sample protein concentration was calculated by substituting the sample absorbance. These data supported the use of sample dilution for subsequent experiments (e.g., Olink detection).

#### 2.3.3 Protein quantification and QC system

The samples were diluted to 1 μg/μl for Olink analysis using the inflammation panel as previously described (9, 15–17). For detailed experimental procedures of the Olink experiment and internal controls of the QC system, please refer to **Supplementary Table 1**.

### 2.4 Bioinformatic analysis

Gene Ontology (GO) analysis of the selected differentially expressed proteins (DEPs) was analyzed in the GO database. Pathway analysis and interaction analysis were implemented by IPA (version 24.0.1) with *P* < 0.05 and a Z score > 0 or < 0 (18, 19).

### 2.5 Statistical analysis

A Student’s t test was performed for the comparison of a pair of groups, and a *p* value < 0.05 was chosen to indicate statistical significance, according to published studies (20). The selection criteria for the DEPs for bioinformatic analysis were a *P* < 0.05 and an FCH ≥1.2 (13, 20). Statistical analysis was performed using R software (version 4.0.1).

## 3 Results

We enrolled 16 adult patients with SSD and 12 HCs. There were no significant differences in age, sex, sleep duration or sleep quality between SSD patients and HCs (**Table 1**). The age distribution of the patients with SSD is shown in **Supplementary Table 2**. Among the 92 markers, 60 markers were detected in the lesional scalps of SSD patients (**Supplementary Table 3**). There was no significant difference between the SSD group and the HC (**Supplementary Figure 1**). Principal component analysis demonstrated that lesional skin of SSD patients clustered separately from that of healthy controls (**Supplementary Figure 2**).

**Table 1:** Demographics of patients with scalp seborrheic dermatitis and HCs.

Characteristics	Scalp seborrheic dermatitis (n=16)	Healthy controls (n=12)	P value
Age (y)			
Mean (SD)	45.4(11.6)	40.8(14.4)	0.37
Range	27-60	24-68	
Sex, n (%)			
Male	9(56.3)	7(58.3)	1.0
Female	7(43.8)	5(41.7)	
Sleep time			
Mean (SD)	7.4(1.0)	7.5(0.9)	0.74
Range	6-9	6-9	
Sleep Quality			
Normal	8(50.0)	9(75.0)	0.25
Poor	8(50.0)	3(25.0)	

Quantitative comparisons were evaluated using Student’s t tests. Analogous comparisons were assessed with Fisher’s exact tests.

### 3.1 The proteomic profiles of SSD patients have increased T-cell/lymphocyte activation

**Figure 1:**
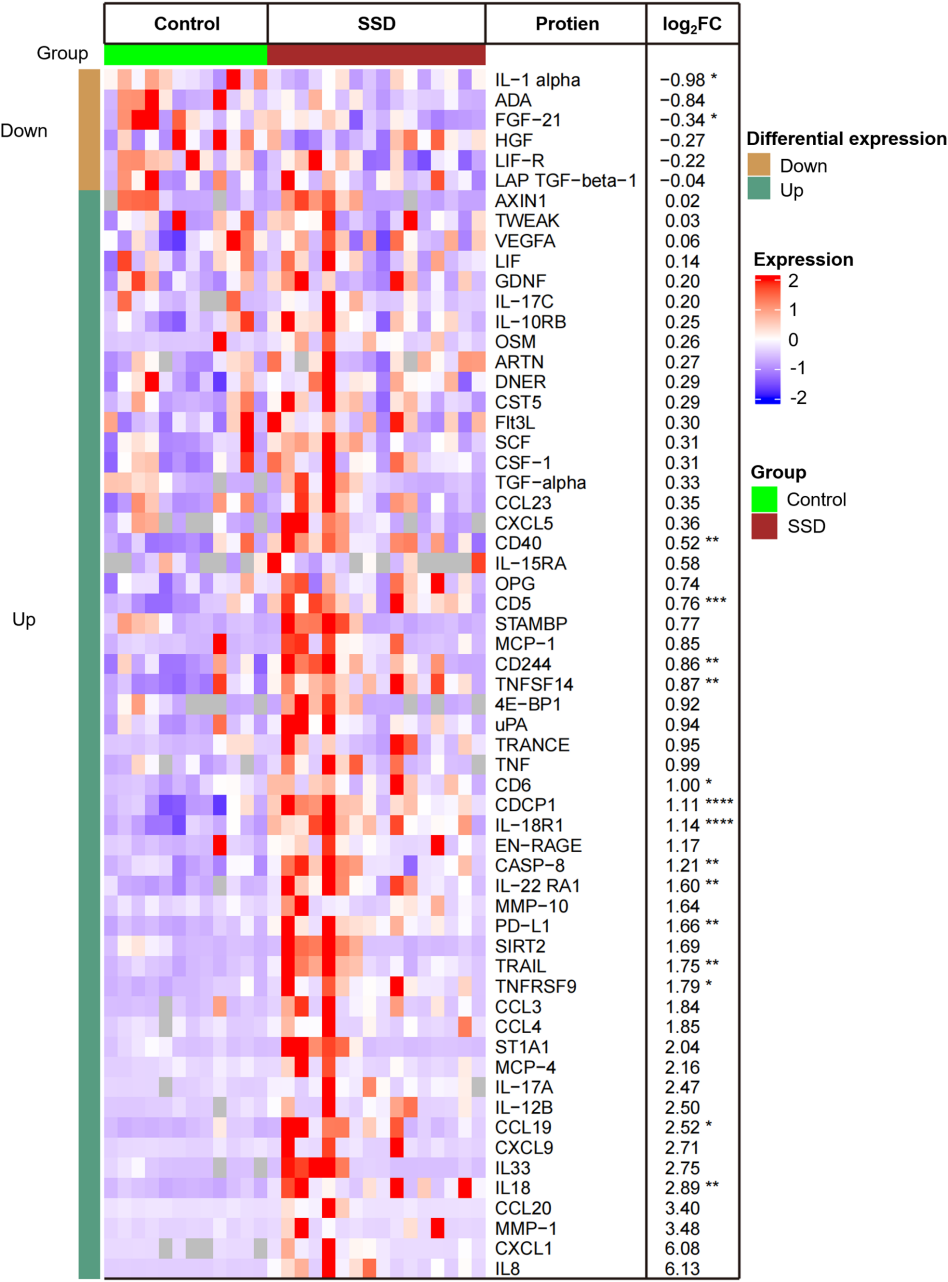
Heatmap of 16 differentially expressed proteins (DEPs) in lesional scalps of patients with SSD compared to with those of healthy controls (HCs). Each column represents an individual patient. Abbreviations: L, lesional scalp; C, healthy controls. The intensity of the colors reflects the degree of change in expression. The right table provides a list of the biomarkers along with their respective FCH values in SSD patients vs. HCs. *P <.05; **P <.01; ***P <.001; ****P <.0001.

Using the criteria of |FCH|≥1.2 and p ≤ 0.05, we identified 16 DEPs in the lesional scalps of SSD patients compared with those of HCs (**Figure 1**). The expressions of IL-1a and FGF-21 were downregulated, while the other fourteen proteins were upregulated. Notably, the expressions of Th1-related markers, such as IL-18 and IL-18R1, were upregulated. IL-8, also known as CXCL8, had greater expression in SSD patients compared with that in the control group, but the difference was not statistically significant, although the P value was close to 0.05.

**Figure 2:**
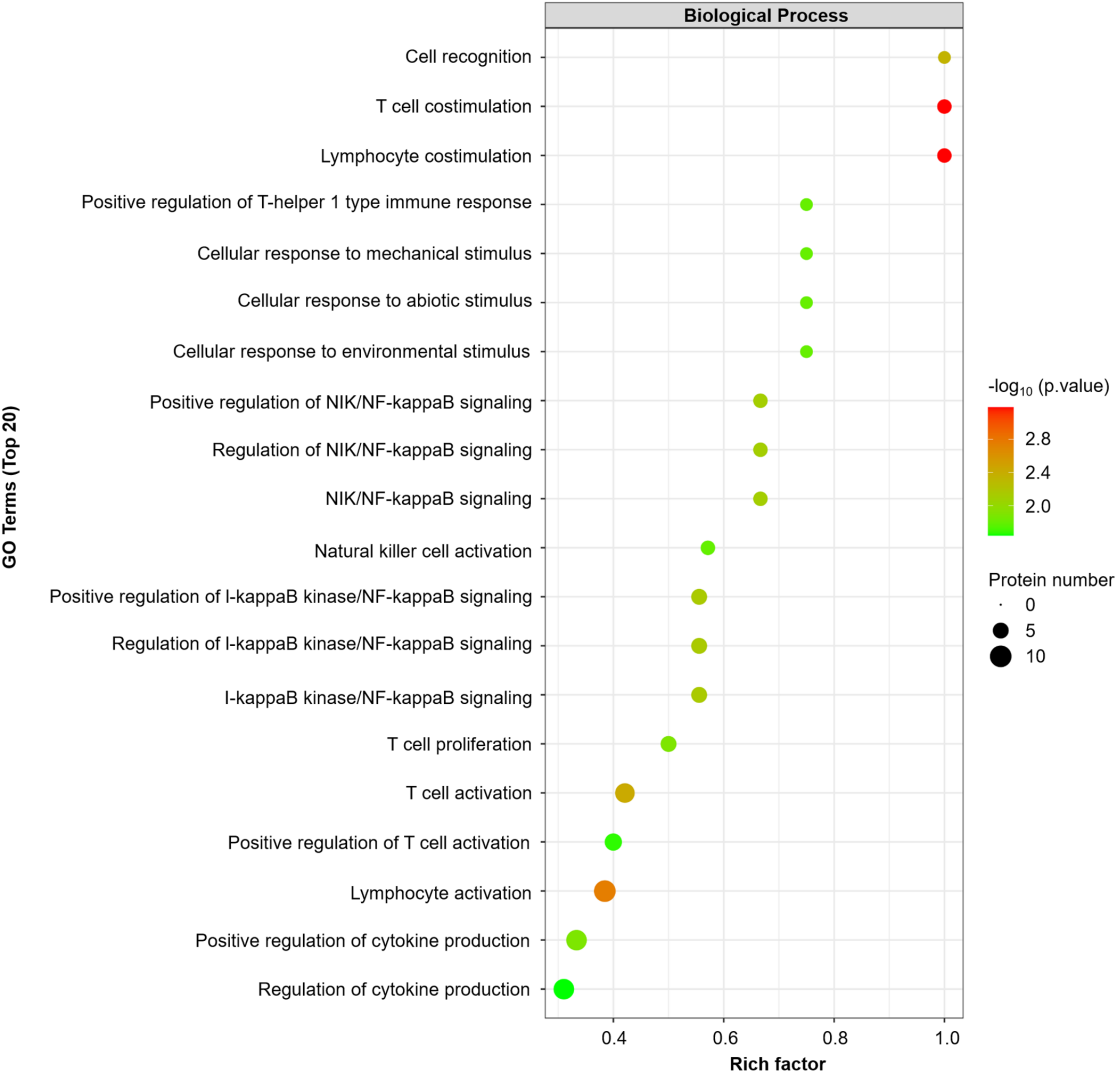
GO enrichment analyses were performed for 16 DEPs (FCH≥ ± 1.2, *P* value<0.05). The dot plot displays the top 20 significantly different GO terms. A *p* value <0.05 was used for biological process selection.

GO enrichment analysis revealed that T-cell/lymphocyte activation was significantly upregulated in lesional skin (**Figure 2**). The involved DEPs were CCL19, CD6, CD244, IL-18, IL-18R1, CASP-8, TNFSF14, PD-L1, CD5, and CD40 (**Supplementary Table 4**). Additionally, the expressions of some molecules (e.g., IL-8, IL-12 and TNF), which have been reported to increase in previous studies of patients with SD, were also upregulated but were not significantly different from those in HCs. Interestingly, the expression of IL-1α was downregulated in SSD patients, which is inconsistent with the findings of prior research.

### 3.2 The protein interaction network revealed that DEPs were involved mainly in the pathogen-induced cytokine storm signaling pathway

**Figure 3:**
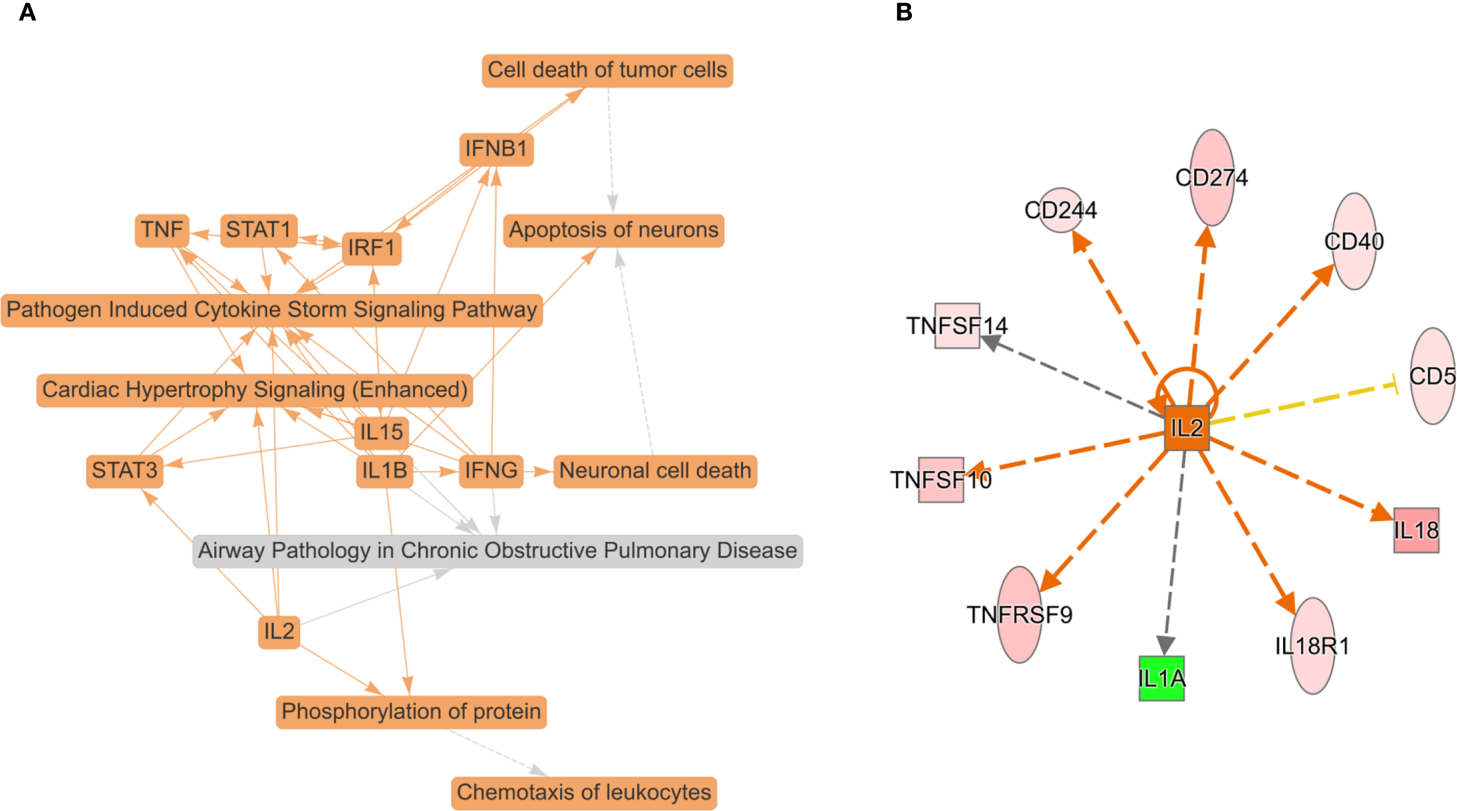
Top biological themes in the network **(A)** and upstream regulator analysis (URA) of 16 DEPs (FC ≥ ± 1.2, *P* value < 0.05) **(B)** according to Z scores determined using IPA (P < 0.05, Z score > 0 or < 0). IPA determines likely upstream regulators that are connected to dataset genes through a set of direct or indirect relationships. The top potential upstream upregulator was IL-2. The green color for protein names indicates downregulation, and red indicates upregulation. The darker the color is, the more significant the change. The relationships among molecules are represented by lines (solid lines for direct associations and dotted lines for indirect associations).

To obtain a systematic understanding of the synergetic networks of DEPs in SSD, we performed network analysis using IPA (absolute [Z score] ≥0). The top biological theme in the network was pathogen induced cytokine storm signaling pathway (**Figure 3A**). The network revealed multiple proteins (IFNB1, IFNG, IL-15, IL-1B, IL-2, IRF1, STAT1, STAT3, and TNF) involved in the pathogenesis-induced cytokine storm signaling pathway and the CGAS-STING signaling pathway. Our data indicate the pivotal role of cytokine signaling in the immune response to pathogen invasion, leading to a potentially severe inflammatory reaction known as a cytokine storm. Further upstream regulatory factor analysis revealed that IL-2 is the central upstream regulator (**Figure 3B**).

### 3.3 Pathway analysis highlights Th1 skewing

To examine the functional pathways of SSD proteomics, we performed a pathway enrichment analysis using IPA (absolute Z score ≥0) (**Supplementary Figure 3**). According to our data, the Th1 pathway was predicted to be activated, and the representative proteins were CCL19, IL-18 and IL-18R1. We observed that Th2-related cytokines, including IL-4, IL-5, IL-10, IL-10RA, and IL-13, were rarely or not detected in SSD patients or HCs because they were below the LOD (**Supplementary Table 5**). Notably, one of the metabolism-related pathways, farnesoid X receptor (FXR)/RXR activation, was predicted to be inhibited.

## 4 Discussion

In this Olink high-throughput proteomics study, we depicted the molecular profiles of SSD patients through a minimally invasive 3 mm trephine. To our knowledge, this is the first study to describe the immunological characteristics of patients with SSD using Olink proteomics. We observed an increased T-cell/lymphocyte activation, CGAS-STING signaling pathway and Pathogen Induced Cytokine Storm Signaling Pathway activation, and skewing of Th1 activation, which is slightly different from the etiology of SSD described in the previous research.

SD is related to the interplay between Malassezia dysbiosis and immune and lipid secretion, but their relationships have not yet been elucidated (5, 21, 22). Sebocytes play a vital role in the interplay of skin immunology and microbiology (23), notably through proinflammatory cytokines (such as IL-1b, IL-8, and TNF-a) in response to fatty acids and reactive oxygen species produced by Malassezia spp (23, 24). Consistent with these studies, our results revealed multiple DEPs involved in the pathogenesis-induced cytokine storm signaling pathway and the CGAS-STING signaling pathway, indicating the immune response to pathogen invasion. Benjamin Ungar et al. (7) carried out a transcriptomic study and reported increased levels of IL-23/Th17/Th22-related markers (such as IL-23, IL-17, IL-36, and IL-22) and Th1-related IL-1b in in individuals with SD. Previous studies have demonstrated that Malassezia can promote Th17 polarization and Th1-related cytokine expression (24–26) and that there is an association between IL-17 and SD (25, 27). In line with the above research, our proteomics study revealed that the DEGs caused an increased T-cell/lymphocyte activation and were enriched mainly in Th1 pathways; however, our data revealed a lack of Th17-related markers (IL-17A and IL-17C) in SSD patients. Different results might be generated by the diverse technical methods, differences between proteins and their corresponding mRNAs (28), and diverse sample types or sampling sites.

We identified several important Th1-related proteins (IL-18, IL-18R1 and IL-1α) that belong to the IL-1 family. IL-18 is a proinflammatory cytokine that is involved primarily in epithelial barrier repair and polarized Th1 cell and natural killer (NK) cell immune responses (29). Upon binding to IL-18R1 and IL-18RAP (30), it forms a complex, triggering the synthesis of inflammatory molecules that positively regulates IL-17 production (31) and activates the NF-kappa-B pathway (32). IL-18 and IL-18R1 were upregulated, suggesting increased T cell/lymphocyte activation, especially in the Th1 cell immune response. Under oily conditions, the expression of the IL-18 gene is upregulated in the response of the skin to *Malassezia sympodialis* (33). These results suggested that IL-18 and IL-18R1 may play important roles in the pathogenesis of SSD. Another IL-1 family member is IL-1α, which binds to its receptor, IL-1R1, to mediate the activation of the NF-kappa-B, MAPK, and JNK pathways (34, 35). Interestingly, the downregulation of IL-1α and the upregulation of IL-18 were inconsistent with prior research (5–7, 22). As we have demonstrated in the protein–protein interaction network, these proteins may be regulated by other molecules or their negative feedback.

Another important finding in our study is the global absence of Th2-related cytokines (such as IL-4, IL-4R, IL-5, IL-10, IL-10RA, TSLP and IL-13) in both SSD patients and healthy individuals. Apart from the influence of technical factors (these proteins were below the LOD), another explanation is that Th2-related cytokines were not dominant in processes of scalp immunity in either the disease state or the nondisease state. These results are consistent with those of an extensive transcriptomic study (16) but inconsistent with those of other studies indicating that Th2-related cytokines were involved in the pathogenesis of SD (3, 27). Sparber et al. (25) also reported that Th2 cytokines (IL-5, IL-13, and TSLP) were downregulated in mice with cutaneous *M. patchy dermatitis* exposure. On the other hand, publications have reported a reaction of SD-like rashes after dupilumab (an IL-4Ra antagonist) treatment (36, 37). Our data suggest that these findings might be caused by further reductions in Th2-related cytokines after blocking IL-4 and IL-13 expression. The expressions of Th2-type cytokines and the role of Th2-type cells in SSD have rarely been studied, and more rigorous research employing multiple verification methods is needed.

In clinical practice, many patients with SSD experience poor sleep. Studies have shown that insufficient sleep can cause more SD and affect sebum secretion (38). However, our research revealed no difference in poor sleep between SSD patients and controls, although the proportion of people with poor sleep was greater in SSD patients than in HCs (50% vs. 25%). Notably, the sleep REM signaling pathway was inhibited. However, the NF-κB pathway was activated in the SSD group, which is consistent with a previous study in which the NF-κB pathway was activated after REM sleep deprivation (39). In *in vitro* and murine models, Malassezia-derived sebum metabolites can promote Th17 polarization and Th1 cytokine expression (24–26).

Furthermore, the dysregulation of lipid metabolism could be caused by the high lipase and phospholipase activities of Malassezia (7, 40–42). Our data revealed that one metabolism-related pathway, farnesoid X receptor (FXR)/RXR activation, was suppressed in the SSD group, indicating lipid regulation dysfunction. Moreover, TSLP is a cytokine that drives the Th2 immune response, but this phenomenon was not detected in this study. Its deficiency at steady state can decrease the production of sebum and antimicrobial peptides and reduce the ability to regulate homeostatic sebum production and skin barrier function (23). In conclusion, the causal relationship between metabolic disorders and pathogen invasion requires further research.

### 4.1 Limitations of the study

(1) The sample size was relatively small. (2) Our analysis was limited to 92 proteins. (3) There was potential for disease misclassification. (4) This cross-sectional study characterized SSD only among adults. (5) GO enrichment analysis was performed for only 16 DEPs. Hence, the associated pathways might have only a few leading-edge proteins contributing to the pathway.

## 5 Conclusions

Overall, this study identified the adult SSD proteomic signature in skin biopsies. Further longitudinal analyses are needed, and unique profiles in patients with immunodeficiency, contributions of Malassezia to SSD, and nonlesional scalps should be examined. Our data suggested that SSD is characterized by increased T-cell/lymphocyte activation, and the skewing of Th1 activation. Our research provides new ideas for clinical treatment. The proteomic scalp profile can be valuable for future studies requiring biomarker monitoring and has application prospects in dermatological diseases (for example, the differential diagnosis between seborrheic dermatitis and scalp psoriasis) because the Olink platform requires as little as 10 µg of tissue, which can be easily acquired through as little as a 1 mm punch biopsy.

## Data Availability

The datasets presented in this study can be found in online repositories. The names of the repository/repositories and accession number(s) can be found in the article/**Supplementary Material**.
