# Supplementary material for: Scalp seborrheic dermatitis demonstrates a skewing of Th1 activation: a proteomic study in lesional skin
Source: Front Immunol. 2025 Sep 29;16:1638710. doi: 10.3389/fimmu.2025.1638710 (PMC12515636; doi:10.3389/fimmu.2025.1638710)
Supplement: Supplementary file 1 [file DataSheet1.docx]

**SUPPLEMENTARY MATERIAL**

**Supplementary Table 1:** Detailed experimental procedures of the Olink experiment and internal controls of the QC system

**1. Incubation Procedure**

1.1 Incubation Mix Preparation

Equilibrate reagents to 20–25°C. Prepare mix in a sterile microcentrifuge tube as per Table 1.1

**Table 1.1 Composition of the Incubation Mix for a Single 96-Well Plate**

| Incubation mix | Per 96-well plate (µL) |
| --- | --- |
| Olink® Target 96 Incubation Solution | 280 |
| Olink® Target 96 Incubation Stabilizer | 40 |
| Olink® Target 96 A-probes | 40 |
| Olink® Target 96 B-probes | 40 |

Total 400

1.2 Dispensing to 8-Well Strips

Vortex mix for 15–30s, centrifuge at 300–500×g for 10s. Transfer 47µL to each well of sterile 8-well strips using a calibrated pipette.

1.3 Incubation Plate Preparation

Label a 96-well PCR plate. Transfer 3µL of mix from 8-well strips to each well using reverse pipetting.

1.4 Sample and Control Loading

Add 1µL sample to each well bottom with a multichannel pipette. For QC (Column 12): 3 wells with 1µL Negative Control, 3 wells with 1µL Interplate Control, and 2 replicate wells with 1µL pooled plasma.

1.5 Sealing, Centrifugation and Incubation

Seal plate with heat-resistant film, centrifuge at 400–1000×g for 1min at RT. Incubate at 4°C overnight (16–18h) for hybridization.

**2. Extension Procedure**

2.1 Extension Mix Preparation

Prepare in a sterile 50mL tube as per Table 2.1

**Table 2.1 Composition of the Extension Mix for a Single 96-Well Plate**

| Extension mix | Per 96-well plate (µL) |
| --- | --- |
| High Purity Water | 9374 |
| Olink® Target 96 PEA Solution | 1100 |
| Olink® Target 96 PEA Enzyme | 55 |
| Olink® Target 96 PCR Polymerase | 33 |

## Total 10 562

Equilibrate reagents to 20–25°C and invert 3–5 times.

2.2 Plate and PCR Machine Preparation

Equilibrate incubation plate to RT for 10–15min, then centrifuge at 400–1000×g for 1min. Preheat thermal cycler to 50°C.

2.3 Extension Mix Preparation for Dispensing

Vortex mix at 1500rpm for 20s, centrifuge at 300×g for 10s. Transfer to a multichannel pipette reservoir.

2.4 Extension Mix Dispensing

Dispense 96µL to upper inner wall of each well using a 12-channel pipette with reverse pipetting.

2.5 Sealing, Mixing and Centrifugation

Seal with new film, vortex at 2000rpm for 30s (verify homogeneity), centrifuge at 400–1000×g for 1min at RT.

2.6 Thermal Cycler Program

Run PEA program with lid at 105°C: 50°C for 20min → 95°C for 5min → 17 cycles (95°C/30s → 54°C/1min → 60°C/1min) → hold at 10°C.

**3. Detection**

Prepare Olink® 96.96 IFC: inject control fluid into accumulators, prime in Q100 instrument.

Thaw, vortex and briefly centrifuge Primer Plate.

Prepare Detection mix (Total for 1×96-well plate: 790.5µL: 550µL Detection Solution, 228µL water, 7.8µL Detection Enzyme, 4.7µL PCR Polymerase).

Vortex, centrifuge, and add 95µL to each 8-well strip well.

Transfer 7.2µL to a new 96-well "Sample Plate" using reverse pipetting.

Centrifuge incubation plate, transfer 2.8µL to corresponding Sample Plate wells.

Seal, vortex, and centrifuge Sample Plate at 400–1000×g for 1min at RT.

Transfer 5µL from Primer Plate to IFC left inlets and 5µL from Sample Plate to right inlets (reverse pipetting, change tips).

Remove bubbles, load chip into Q100, follow instrument prompts.

Run detection with appropriate interface plate.

**QC system**

Internal controls

The QC system consists of four internal controls that are spiked into every sample and are designed to monitor the three main steps of the Olink protocol: Immunoreaction, extension and amplification/detection.

Incubation controls: Incubation Control 1 and 2 are two different non-human antigens measured with PEA. These controls monitor potential technical variation in all three steps of the reaction.

Extension control: The Extension Control is composed of an antibody coupled to a unique pair of DNA-tags. These DNA-tags are always in proximity, so that this control is expected to give a constant signal independently of the immunoreaction. This control monitors variation in the extension and amplification/detection step and is used to adjust the signal from each sample with respect to extension and amplification.

Detection control: The Detection Control is a complete double stranded DNA amplicon which does not require any proximity binding or extension step to generate a signal. This control monitors the amplification/detection step.

**Supplementary Table 2:** The age distribution of the patients with SSD.

| Patient Number | Age |
| --- | --- |
| DYH | 57 |
| FZC | 52 |
| HGX | 51 |
| JQF | 27 |
| LLD | 56 |
| LPC | 49 |
| LXL | 48 |
| MZG | 27 |
| QYM | 36 |
| RBX | 60 |
| SWX | 55 |
| WL | 29 |
| WZM | 54 |
| XDJ | 34 |
| YFX | 37 |
| YGB | 54 |

**Supplementary Table 3:** 60 of 92 detected markers in lesional scalp of SSD compared to the controls.

**Supplementary Table 4:** Top 20 GO terms in lesional scalp of SSD compared to the controls.

| Term | Test | TestAll | Test_per | TestSeqs | P value | richFactor |
| --- | --- | --- | --- | --- | --- | --- |
| T cell costimulation | 4 | 16 | 0.25 | O43557,Q99731,Q9NZQ7,P06127 | 0.00065136 | 1 |
| lymphocyte costimulation | 4 | 16 | 0.25 | O43557,Q99731,Q9NZQ7,P06127 | 0.00065136 | 1 |
| lymphocyte activation | 10 | 16 | 0.625 | Q99731,P30203,Q9BZW8,Q14116,Q13478,Q14790,P25942,O43557,Q9NZQ7,P06127 | 0.001773322 | 0.384615385 |
| T cell activation | 8 | 16 | 0.5 | Q99731,O43557,Q9NZQ7,P06127,P30203,Q14116,Q13478,Q14790 | 0.003753335 | 0.421052632 |
| cell recognition | 3 | 16 | 0.1875 | P30203,Q99731,P06127 | 0.004459309 | 1 |
| I-kappaB kinase/NF-kappaB signaling | 5 | 16 | 0.3125 | P50591,P01583,Q99731,P25942,Q14790 | 0.00713733 | 0.555555556 |
| regulation of I-kappaB kinase/NF-kappaB signaling | 5 | 16 | 0.3125 | P50591,P01583,Q99731,P25942,Q14790 | 0.00713733 | 0.555555556 |
| positive regulation of I-kappaB kinase/NF-kappaB signaling | 5 | 16 | 0.3125 | P50591,P01583,Q99731,P25942,Q14790 | 0.00713733 | 0.555555556 |
| NIK/NF-kappaB signaling | 4 | 16 | 0.25 | Q14116,O43557,Q99731,Q13478 | 0.007750976 | 0.666666667 |
| regulation of NIK/NF-kappaB signaling | 4 | 16 | 0.25 | Q14116,O43557,Q99731,Q13478 | 0.007750976 | 0.666666667 |
| positive regulation of NIK/NF-kappaB signaling | 4 | 16 | 0.25 | Q14116,O43557,Q99731,Q13478 | 0.007750976 | 0.666666667 |
| T cell proliferation | 5 | 16 | 0.3125 | P30203,Q99731,Q9NZQ7,Q14116,O43557 | 0.012733075 | 0.5 |
| positive regulation of cytokine production | 9 | 16 | 0.5625 | P01583,Q14116,Q13478,Q14790,Q99731,P25942,P30203,Q9BZW8,Q9NZQ7 | 0.012940588 | 0.333333333 |
| cellular response to mechanical stimulus | 3 | 16 | 0.1875 | O43557,P25942,Q14790 | 0.015883156 | 0.75 |
| positive regulation of T-helper 1 type immune response | 3 | 16 | 0.1875 | Q99731,Q14116,Q13478 | 0.015883156 | 0.75 |
| cellular response to environmental stimulus | 3 | 16 | 0.1875 | O43557,P25942,Q14790 | 0.015883156 | 0.75 |
| cellular response to abiotic stimulus | 3 | 16 | 0.1875 | O43557,P25942,Q14790 | 0.015883156 | 0.75 |
| natural killer cell activation | 4 | 16 | 0.25 | Q9BZW8,Q14116,Q13478,Q14790 | 0.016096567 | 0.571428571 |
| positive regulation of T cell activation | 6 | 16 | 0.375 | O43557,Q99731,Q9NZQ7,P06127,P30203,Q14116 | 0.021176034 | 0.4 |
| regulation of cytokine production | 9 | 16 | 0.5625 | P01583,Q9NZQ7,Q14116,Q13478,Q14790,Q99731,P25942,Q9BZW8,P30203 | 0.022768681 | 0.310344828 |

**Supplementary Table 5:** The NPX and LOD value of five Th2-related cytokines.

| Sample number | Panel | Olink Target 96 Inflammation (v.3027) | | | | | | |
| --- | --- | --- | --- | --- | --- | --- | --- | --- |
|  | Assay | IL4 | IL5 | IL10 | IL-10RA | IL13 | Plate ID | QC Warning |
|  | Uniprot ID | P05112 | P05113 | P22301 | Q13651 | P35225 |  |  |
|  | OlinkID | OID00546 | OID00559 | OID00528 | OID00508 | OID00525 |  |  |
|  |  |  |  |  |  |  |  |  |
| DYH_L |  | -1.98032 | 0.90676 | -0.21403 | -0.22840 | 0.15191 | INF-20250314-1 | Pass |
| FZC_L |  | -0.09090 | -0.07990 | -0.31038 | -0.36054 | 1.50227 | INF-20250314-1 | Pass |
| HGX_L |  | -1.13493 | -0.17594 | -0.70335 | -0.44972 | 2.26375 | INF-20250314-1 | Pass |
| JQF_L |  | -2.06845 | -0.14613 | -0.39578 | -0.88665 | -0.86556 | INF-20250314-1 | Pass |
| LLD_L |  | -1.39697 | 0.30046 | 0.27451 | -0.41132 | 1.16319 | INF-20250314-1 | Pass |
| LPC_L |  | -1.35992 | 0.59926 | -0.10477 | -0.53768 | 0.24633 | INF-20250314-1 | Pass |
| LXL2_L |  | -1.15979 | -0.24252 | -0.63956 | -1.16009 | 0.25737 | INF-20250314-1 | Pass |
| MZG_L |  | -1.52872 | -0.61491 | -0.59655 | -0.61539 | -0.42529 | INF-20250314-1 | Warning |
| QYM_L |  | -1.49077 | -0.02180 | -0.26467 | -1.02103 | -0.65009 | INF-20250314-1 | Warning |
| RBX_L |  | -1.06069 | 0.28699 | -0.32208 | -0.64152 | 0.30906 | INF-20250314-1 | Pass |
| SWX_L |  | -2.08155 | -0.00965 | -0.89554 | -0.37434 | 1.52109 | INF-20250314-1 | Pass |
| WL_L |  | -1.53398 | 0.20478 | -0.39559 | -0.89858 | -0.52824 | INF-20250314-1 | Pass |
| WZM_L |  | -2.01571 | -0.72941 | 0.12106 | -0.72902 | -0.18387 | INF-20250314-1 | Pass |
| XDJ_L |  | -0.73856 | 0.24881 | -1.49227 | -0.92981 | -0.57653 | INF-20250314-1 | Pass |
| YFX_L |  | -2.18758 | -0.50677 | -0.77704 | -0.35572 | 0.20984 | INF-20250314-1 | Warning |
| YGB_L |  | -2.46846 | -0.29611 | -0.83312 | -1.00539 | -1.03638 | INF-20250314-1 | Warning |
| CJX_C |  | -1.48591 | 0.04477 | -0.92698 | -0.60302 | -0.67522 | INF-20250314-1 | Warning |
| CML_C |  | -2.36072 | 0.99951 | -0.03078 | -0.75867 | -0.06296 | INF-20250314-1 | Warning |
| HX_C |  | -1.13264 | 1.38581 | 0.25106 | -0.28566 | 0.09084 | INF-20250314-1 | Pass |
| LSJ_C |  | -1.20235 | 1.64711 | 0.61894 | -0.47232 | 0.29326 | INF-20250314-1 | Warning |
| LJM_C |  | -1.47514 | 0.97254 | -0.60437 | -0.56615 | 0.28447 | INF-20250314-1 | Pass |
| LXW_C |  | -1.74385 | 0.44603 | -0.56130 | -1.39581 | 0.04384 | INF-20250314-1 | Pass |
| PGM_C |  | -1.40892 | -0.04972 | -0.58835 | -1.26158 | 0.18949 | INF-20250314-1 | Warning |
| XQ_C |  | -2.56966 | 0.35217 | -0.19328 | -0.79081 | -0.19852 | INF-20250314-1 | Warning |
| XWH_C |  | -2.09414 | -0.38164 | -1.10550 | -1.26100 | -0.93221 | INF-20250314-1 | Pass |
| ZZH_C |  | -1.33668 | 0.16221 | -1.04491 | -1.24387 | -0.56065 | INF-20250314-1 | Warning |
| ZXF_C |  | -1.58763 | -0.45139 | -0.78272 | -0.95546 | -0.20056 | INF-20250314-1 | Pass |
| ZZP_C |  | -0.61779 | -0.06764 | -0.67437 | -0.87587 | -0.10314 | INF-20250314-1 | Pass |
|  | MaxLOD | -0.86228 | 0.68785 | 0.38030 | -0.14149 | 0.80703 |  |  |
|  | PlateLOD | -0.86228 | 0.68785 | 0.38030 | -0.14149 | 0.80703 | INF-20250314-1 | |
|  | Missing Data freq. | 89% | 82% | 96% | 100% | 91% |  |  |
|  | Normalization | IPC Normalized | IPC Normalized | IPC Normalized | IPC Normalized | IPC Normalized | |  |

**Supplementary Figure 1:** Comparison of protein identification quantities of Seborrheic Seborrheic Dermatitis compared to healthy controls.


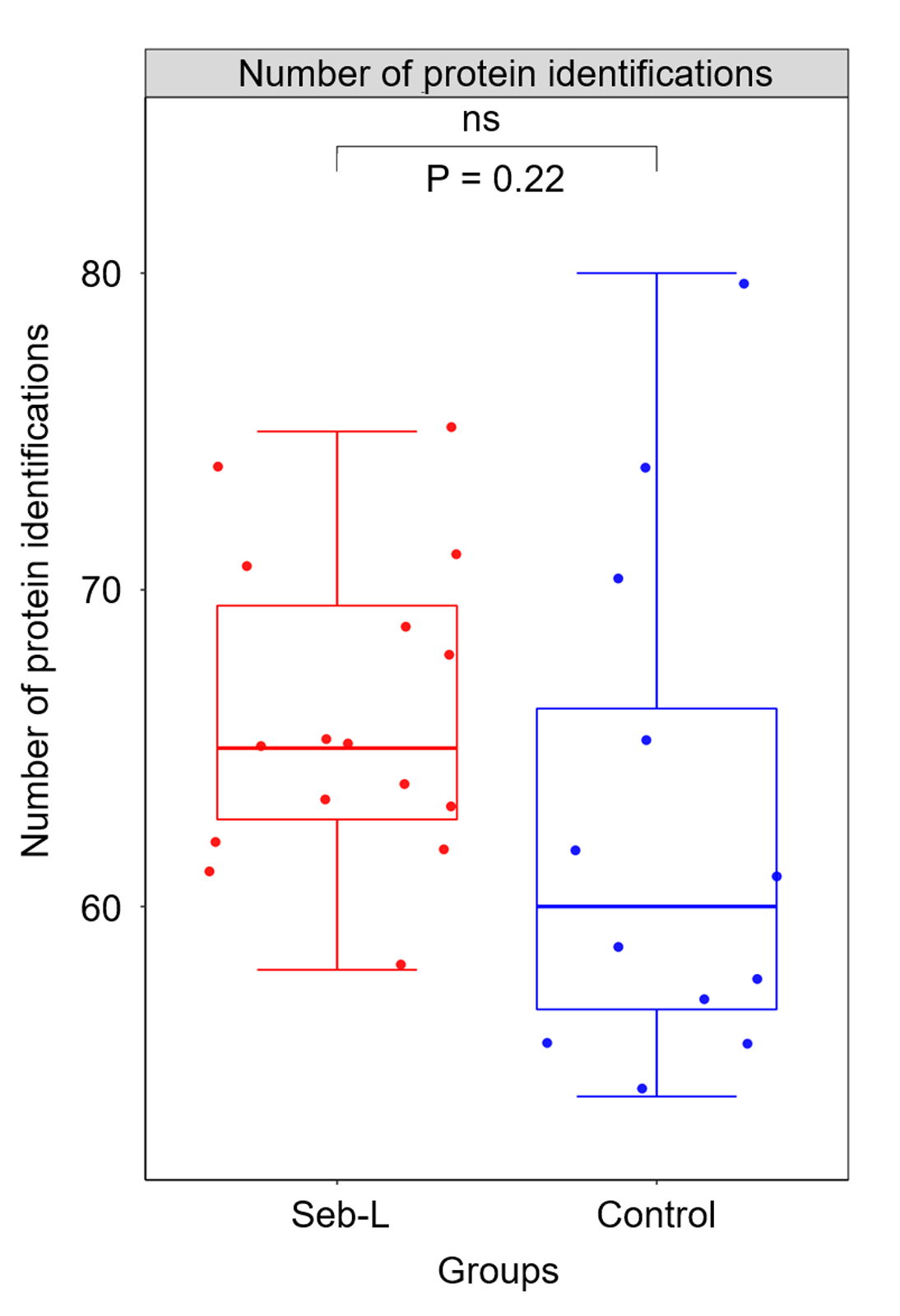


**Supplementary Figure 2:** Principal component analysis of Seborrheic Seborrheic Dermatitis compared to healthy controls.


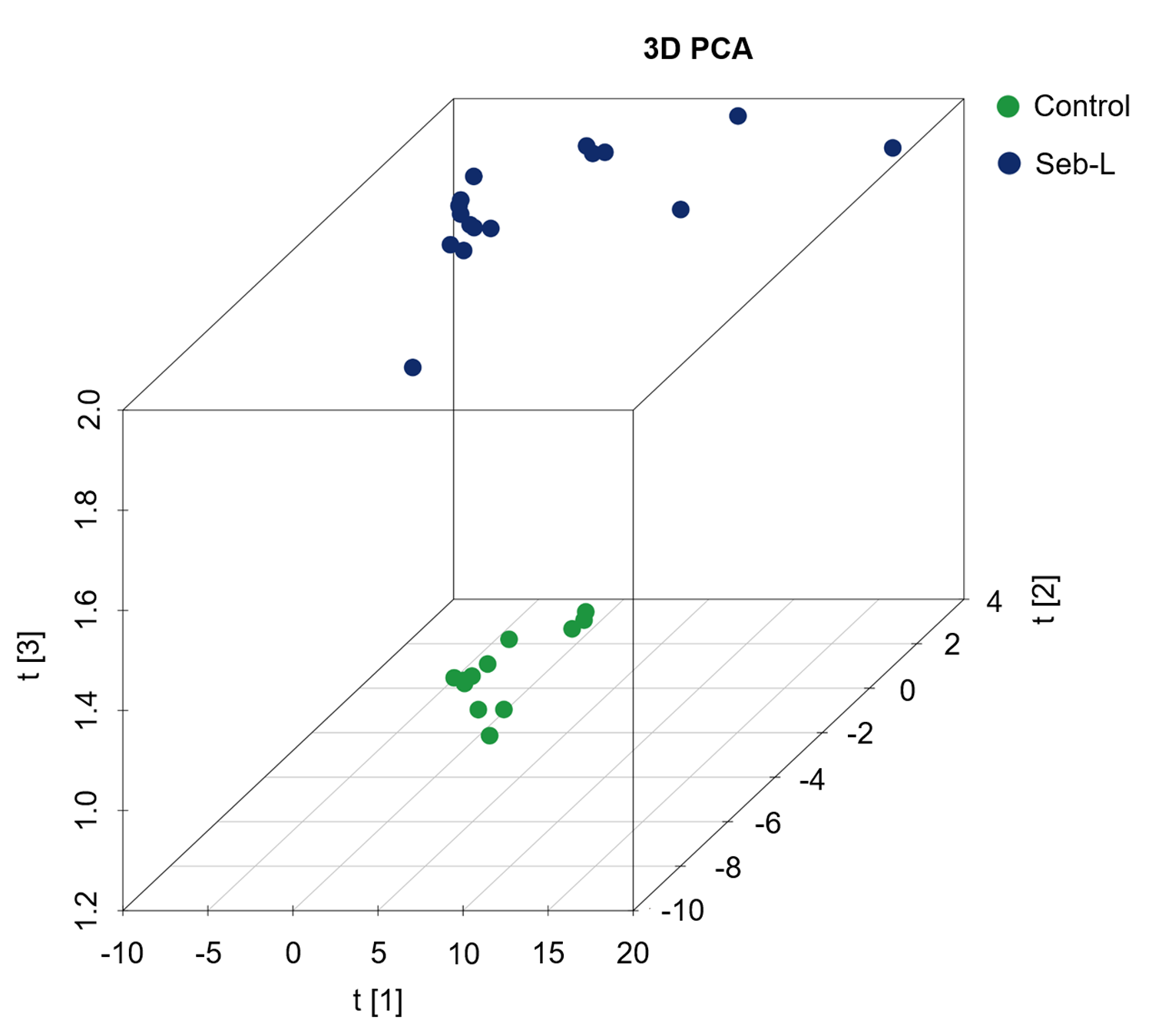


**Supplementary Figure 3:** TOP 22 pathways in lesional scalp group, compared to HCs group. Ingenuine pathway analysis (IPA) was performed for pathway analysis. The color represents the Z score by IPA and the predicted activation (orange) or suppression (blue) state. The darker the color, the more significant the change.

**
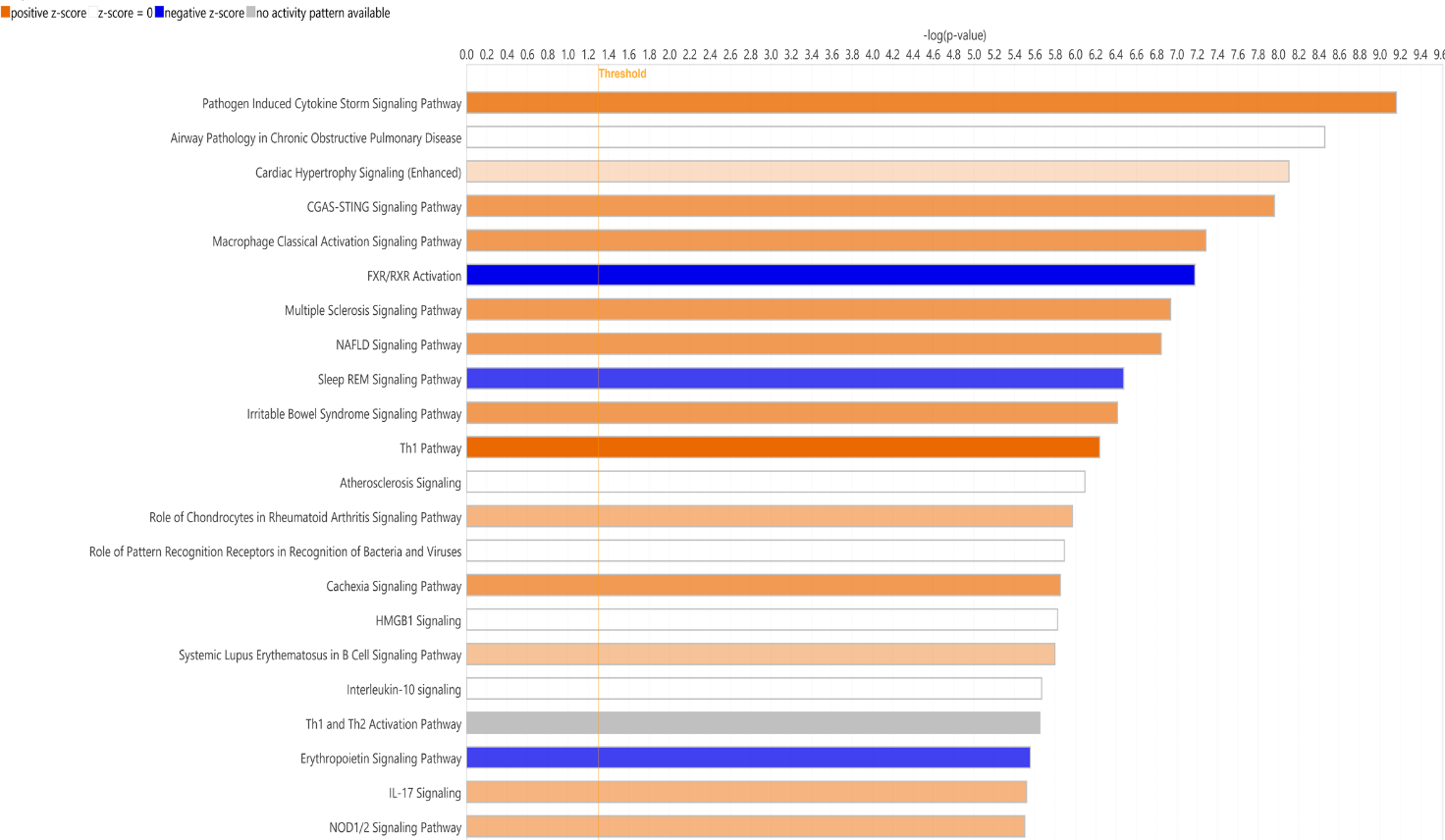
**
